# Influence of naturally-occurring 5′-pyrophosphate-linked substituents on the binding of adenylic inhibitors to ribonuclease a: An X-ray crystallographic study

**DOI:** 10.1002/bip.21158

**Published:** 2009-02-03

**Authors:** Daniel E Holloway, Gayatri B Chavali, Demetres D Leonidas, Matthew D Baker, K Ravi Acharya

**Affiliations:** Department of Biology and Biochemistry, University of BathClaverton Down, Bath BA2 7AY, UK

**Keywords:** enzyme inhibitor, RNase A, angiogenin, eosinophil-derived neurotoxin, ATP, diadenosine polyphosphates, NADPH, NADP^+^, pyrophosphate

## Abstract

Ribonuclease A is the archetype of a functionally diverse superfamily of vertebrate-specific ribonucleases. Inhibitors of its action have potential use in the elucidation of the in vivo roles of these enzymes and in the treatment of pathologies associated therewith. Derivatives of adenosine 5′-pyrophosphate are the most potent nucleotide-based inhibitors known. Here, we use X-ray crystallography to visualize the binding of four naturally-occurring derivatives that contain 5′-pyrophosphate-linked extensions. 5′-ATP binds with the adenine occupying the B_2_ subsite in the manner of an RNA substrate but with the γ-phosphate at the P_1_ subsite. Diadenosine triphosphate (Ap_3_A) binds with the adenine in *syn* conformation, the β-phosphate as the principal P_1_ subsite ligand and without order beyond the γ-phosphate. NADPH and NADP^+^ bind with the adenine stacked against an alternative rotamer of His119, the 2′-phosphate at the P_1_ subsite, and without order beyond the 5′-α-phosphate. We also present the structure of the complex formed with pyrophosphate ion. The structural data enable existing kinetic data on the binding of these compounds to a variety of ribonucleases to be rationalized and suggest that as the complexity of the 5′-linked extension increases, the need to avoid unfavorable contacts places limitations on the number of possible binding modes. © 2009 Wiley Periodicals, Inc. Biopolymers 91: 995–1008, 2009.

This article was originally published online as an accepted preprint. The “Published Online” date corresponds to the preprint version. You can request a copy of the preprint by emailing the Biopolymers editorial office at biopolymers@wiley.com

## INTRODUCTION

Bovine pancreatic ribonuclease A (RNase A; EC 3.1.27.5) has long been studied as a model of enzyme structure and function (reviewed by Raines^1^). It is now increasingly recognised as the archetype of an intriguing enzyme superfamily whose actions are closely linked to the evolution of vertebrates and which has undergone tremendous diversification.^2^–^5^ Many members of the superfamily have potent biological activities that are dependent on their enzymatic activity. Among these are the angiogenic and tumor-promoting activities of angiogenin (Ang),^6^–^8^ the antiviral and neurotoxic activities of eosinophil-derived neurotoxin (EDN)^9^,^10^ and the cytotoxic activities of bovine seminal ribonuclease^11^ and Onconase.^12^ The use of site-directed mutagenesis and broad-spectrum inhibitors such as ribonuclease inhibitor (RI) protein^13^ have been invaluable in establishing the links between biological and enzymatic activities. However, the development of stable, low-molecular weight inhibitors with high specificity remains a priority for the study of individual enzymes in their natural settings. Furthermore, such compounds may lead to agents for the treatment of pathologies associated with ribonuclease action, most notably anti-tumor agents directed at Ang.^8^

RNase A superfamily members cleave the P-O5′ bonds of RNA specifically after pyrimidine residues.^14^ Inhibition of this action with small molecules has focused principally on the central catalytic and substrate-binding region. This is conventionally divided into subsites named P_1_, B_1_, and B_2_, which bind the scissile bond and the heterocyclic bases immediately upstream (a pyrimidine) and downstream (preferentially a purine), respectively.^14^,^15^ One well-established line of attack involves phosphoadenosine-based nucleotides. Such compounds inhibit a variety of ribonucleases including RNase A, EDN, eosinophil cationic protein (ECP), RNase 4, Ang and, in all likelihood, many others.^16^ The tightest-binding examples reported thus far are chemically-synthesized derivatives of adenosine 5′-pyrophosphate (ppA); among these, 5′-diphosphoadenosine 3′-phosphate (ppA-3′-p) and its 5′-phospho 2′-deoxyuridine derivative (pdUppA-3′-p) exhibit *K*_i_ values of 10^−8^–10^−6^*M* when binding to RNase A, EDN, or RNase 4.^17^,^18^ This has been matched only by oligo (vinylsulfonic acid), a polyanion that inhibits RNase A with a *K*_i_ of 0.12 μ*M* under similar buffer conditions containing 0.1*M* NaCl.^19^

X-ray crystallographic studies of complexes formed between phosphoadenosine-based inhibitors and RNase A,^20^–^22^ EDN,^23^,^24^ and ECP^25^ have shown that these compounds bind minimally to the P_1_ and B_2_ subsites but can also make additional interactions further afield depending on the nature of substitution. Exploration of the more peripheral interactions may lead to the development of inhibitors that are specific to particular ribonuclease homologs. However, these enzyme·inhibitor systems exhibit remarkable conformational complexity and the confidence with which inhibitor improvements can be derived from the existing data is not high. For example, with the RNase A·inhibitor system (which has received most attention thus far; Table I), a simple RNA-derived compound such as pA-3′-p binds in the conventional manner observed for oligonucleotide substrate analogues^32^–^35^ (here designated as Class Ia) but a radically altered mode is observed upon modification of the compound with phosphate groups at the 5′- and/or 2′- positions. The two key torsional parameters that characterize this are the rotameric state of His119 (a residue that contributes to both the P_1_ and B_2_ subsites) and the *N*-glycosidic torsion angle (χ) of the nucleotide. These reflect to a large degree the nature of the base-stacking interaction at the B_2_ subsite and the region of the inhibitor that occupies the P_1_ subsite.

**Table I:** Categorized Binding of 5′-Phosphoadenosine-Based Inhibitors to RNase A

	Ring Stacking Partners (B_2_ Subsite)		*N*-glycosidic Torsion Angle, χ^b^	P_1_ Subsite	Representatives			
	His119 Rotamer^a^	Adenine Subfragment	Range in Degrees	Ligand	Compound	PDB Entry	Protein Chain^c^	Reference
Class Ia	A	Pentacyclic ring	293–314 (high-*anti*)	5**′**-P_α_	pA	1Z6S	A(A), B	26
					pA-3**′**-p	1O0F	A, B	22
					pA-2**′**-p	1O0O	B	22
Class Ib	A	Pentacyclic ring	<293 (*anti*/high-*anti*)	Not 5**′**-P_α_	pA	1Z6S	A(B)	26
Class II	A	Hexacyclic ring	31–88 (*syn*)	5**′**-P_β_	ppA	1O0H	A	22
					ppA-3**′**-p	1AFK	A, B	20
					ppA-2**′**-p	1AFL	A, B	20
					dUppA	1JN4	A	27
					pdUppA-3**′**-p	1QHC	A, B	21
					pdTppA-3**′**-p	1U1B	A, B	28
Class III	B	Both rings	277 (high-anti)	2′-P	pA-2′-p	1O0O	A	22

a For rotamer A, χ_1_ ≈ 150° and χ_2_ ≈ −100°; for rotamer B, χ_1_ ≈ −70° and χ_2_ ≈ −60°.^29^

b The *N*-glycosidic torsion angle, χ is the rotation about O4′–C1′–N9–C4. In RNA, χ is usually *anti* (180–250°) or high-*anti* (above 250°) but rarely *syn* (<120°).^30^,^31^

c Letters in parentheses denote alternate ligand conformations.

In view of the conformational uncertainties in the binding of adenylic nucleotides, it remains a priority to extend the panel of inhibitor complexes for which structural data is available. It is pertinent that several naturally-occuring nucleotides that possess a ppA moiety are also effective ribonuclease inhibitors.^36^ These include 5′-ATP, *P*^1^,*P*^3^-bis(5′-adenosyl) triphosphate (Ap_3_A), NADPH, and NADP^+^. We present here the X-ray crystal structures of these compounds in complex with RNase A, along with that of a pyrophosphate ion (PP_i_) complex of the same. They provide new insight into the consequences of a variety of 5′-linked extensions on the binding of adenylic nucleotides to RNase A and its homologs, and indicate how improved inhibitors might be developed.

## RESULTS

### Structural Overview

The structures of monoclinic RNase A crystals soaked with 5′-ATP, Ap_3_A, NADPH, NADP^+^, and PP_i_ were obtained at resolutions of 1.7, 2.4, 1.7, 1.7, and 1.8 Å, respectively, as summarized in Table II. This crystal form presents two protein chains (mol A and mol B) in the asymmetric unit,^20^ and, with the exception of the Ap_3_A complex, which lacks residue LysA1, each of the structures comprises two complete main chains (residues 1–124). Density for residues 16–24 and 87–90 of each chain is consistently poor, as is typical for this crystal form. In Ramachandran plots, 94.2–96.7% of non-Pro/Gly residues lie in the most-favored regions with only SerB16 falling in a disfavored region. In no case is the main chain topology perturbed significantly by the presence of the ligand; of the 10 protein chains, nine have C^α^ traces that deviate by 0.21–0.25 Å (r.m.s.) from the corresponding chain in monoclinic unliganded RNase A (PDB entry 1AFU),^20^ whereas for chain A of the Ap_3_A complex, the figure is slightly higher at 0.48 Å and appears to be a consequence of the relatively low resolution of this structure.

**Table II:** Crystallographic Statistics

	RNase A·5′-ATP	RNase A·Ap_3_A	RNase A·NADPH	RNase A·NADP^+^	RNase A·PP_i_
Diffraction data					
X-ray wavelength (Å)	0.98	1.54	0.87	0.87	0.98
Resolution range					
All data (Å)	40–1.70	40–2.40	40–1.70	40–1.70	40–1.80
Outermost shell (Å)	1.76–1.70	2.49–2.40	1.76–1.70	1.76–1.70	1.86–1.80
Space group	*C*2	*C*2	*C*2	*C*2	*C*2
Unit cell dimensions					
*a* (Å)	101.73	101.96	102.96	102.89	101.33
*b* (Å)	33.27	33.40	33.72	33.70	33.46
*c* (Å)	73.49	75.70	74.15	74.25	73.85
β (deg)	90.10	91.05	89.95	89.97	90.23
No. of reflections					
Measured	64,745	22,719	83,678	156,497	66,478
Unique	26,455	9,496	27,237	27,006	22,438
*R*_symm_^a^^,^^b^	0.078 (0.330)	0.090 (0.337)	0.031 (0.119)	0.042 (0.098)	0.092 (0.354)
*I*/σ (*I*)^b^	10.7 (2.3)	10.8 (2.0)	26.6 (8.1)	21.3 (10.3)	9.5 (4.2)
Completeness (%)^b^	96.1 (95.8)	92.3 (94.6)	95.6 (92.3)	94.8 (88.3)	95.9 (97.1)
Wilson *B*-factor	31.6	39.8	23.4	21.9	30.9
Refined model					
*R*_cryst_^c^	0.198	0.198	0.177	0.185	0.187
*R*_free_^d^	0.230	0.273	0.205	0.227	0.222
Deviation from ideality (r.m.s.)					
Bond lengths (Å)	0.013	0.014	0.014	0.015	0.012
Bond angles (deg)	1.48	1.61	1.38	1.39	1.38
No. of atoms					
Protein (mol A, mol B)	923, 931	907, 919	949, 953	938, 940	943, 940
Ligand (mol A, mol B)	31, 31	31, 31	27, 27	27, 27	9, 9
Water	117	36	171	151	125
Mean *B*-factor (Å^2^)					
Protein (mol A, mol B)	32.5, 28.5	42.6, 34.5	23.1, 21.7	21.5, 20.5	32.2, 27.9
Ligand (mol A, mol B)	39.6, 45.9	50.9, 67.3	32.1, 39.2	28.3, 35.7	31.9, 26.2
Water	38.6	34.0	34.8	32.8	38.3
Ligand occupancy (mol A, mol B)	0.70, 0.70	1.00, 1.00	1.00, 1.00	1.00, 1.00	0.75, 1.00

a *R*_symm_ = Σ_*h*_Σ_*i*_[|*I*_*i*_(*h*) − 〈*I*(*h*)〉|/Σ_*h*_Σ_*i*_*I*_*i*_(*h*)], where *I*_*i*_ is the *i*th measurement and 〈*I*(*h*)〉 is the weighted mean of all measurements of *I*(*h*).

b Values in parentheses refer to the outermost shell.

c *R*_cryst_ = Σ_*h*_|*F*_o_−*F*_c_|/Σ_*h*_*F*_o_, where *F*_o_ and *F*_c_ are the observed and calculated structure factor amplitudes of reflection *h*, respectively.

d *R*_free_ is equal to *R*_cryst_ for a randomly selected 5–10% of reflections not used in the refinement.^37^

**FIGURE 1:**
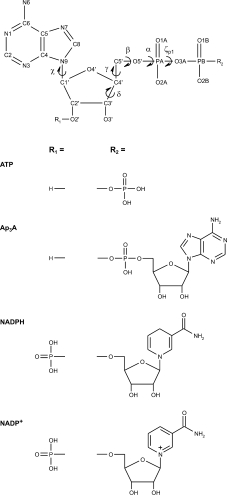
Chemical structures, harmonized atom numbering, and conserved torsion angles of 5′-ATP, Ap_3_A, NADPH, and NADP^+^.

**FIGURE 2:**
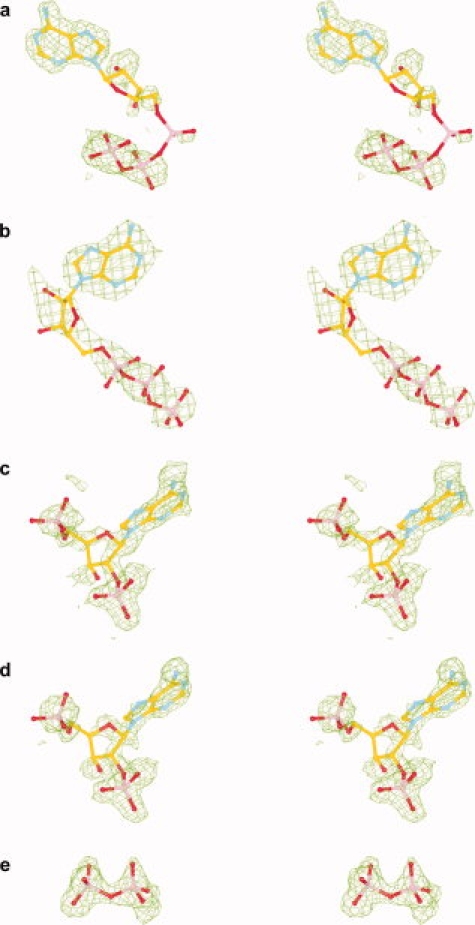
Electron density maps for the nucleotide inhibitors. Shown in stereo for (a) 5′-ATP (mol A), (b) Ap_3_A (mol A), (c) NADPH (mol A), (d) NADP^+^ (mol A), and (e) PP_i_ (mol B) is the ball-and-stick structure of the refined inhibitor (or fragment thereof) superposed with the corresponding portion of the total OMIT map calculated with SFCHECK^62^ and contoured at 1.3σ. The coloring scheme is: carbon, gold; nitrogen, blue; oxygen, red; phosphorus, pink; electron density, green.

Chemical structures, harmonized atom nomenclature, and conserved torsion angles of the nucleotide inhibitors are presented in Figure 1. For each of these compounds, the electron density data indicate clearly the position of one adenine ring and 2–3 phosphate groups but not that of the intervening ribosyl moiety (Figure 2). This parallels the difficulty experienced in locating the ribosyl backbone atoms in many RNA structures.^38^ Plausible coordinates were derived through crystallographic refinement with REFMAC5^39^ with referral to the all-atom contact analysis of MolProbity^40^ and in the knowledge that the lever-actions of the base and phosphates dictate to a large degree the sugar's conformation.^41^ The position of each of the nucleotide inhibitors is better defined in mol A than in mol B. In several cases, this may be due to the stabilizing influence of a crystal contact, as described in more detail below.

### Structure of the RNase A·5′-ATP Complex

**FIGURE 3:**
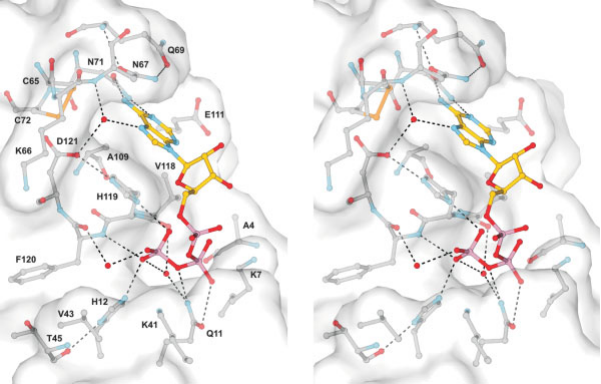
RNase A·5′-ATP complex (mol A). Shown in stereo are ball-and-stick representations of the inhibitor and RNase A residues in and around the binding site, along with a surface representation of the enzyme. Main chain N, C, and O atoms of residues K7, Q11, H12, K41, V43, and E111 are omitted for clarity. The coloring scheme is: nucleotide carbon, gold; protein carbon, gray; nitrogen, blue; oxygen, red; phosphorus, pink. Spheres denote water molecules and dashed lines denote hydrogen bonds.

The inhibition of RNase A by simple 5′-phosphorylated adenosines becomes progressively stronger as the number of phosphates is increased. Under equivalent conditions, *K*_i_ values for 5′-AMP (pA), 5′-ADP (ppA), and 5′-ATP (pppA) are 80, 1.2, and 0.86 μ*M*, respectively.^18^,^36^ The 67-fold drop in *K*_i_ experienced when moving from 5′-AMP to 5′-ADP coincides with a radical change in nucleotide conformation (Class Ia/Ib to Class II; Table I).^22^,^26^ We now present the crystal structure of the RNase A·5′-ATP complex. The electron density map indicates the position of the base, the β- and the γ-phosphate but there is significant disorder in between (Figure 2a). The adenine ring is positioned as in the 5′-AMP complex, stacking with His119 (rotamer A) and hydrogen-bonding with Asn71 in a conventional (Class Ia) fashion, but a small decrease in χ coupled with a pronounced curvature of the triphosphate chain brings the γ- rather than the α-phosphate into the P_1_ subsite (Figure 3; Table III). As such, the binding mode conforms to Class Ib. The two instances of the inhibitor in the asymmetric unit show modest differences in their ribose and triphosphate conformations that permit alternative hydrogen-bonding arrangements. Bonds are consistently made between the γ-phosphate and His12, His119, and Phe120 but involve different inhibitor atoms (Table IV). Also, whereas in mol A there are two potential hydrogen bonds between the β-phosphate and Gln11, in mol B there is one between the γ-phosphate and this residue. There are no direct interactions between the inhibitor and symmetry-related protein atoms that might explain the differences, and the underlying cause is not clear. Water-mediated interactions are made with Gln11, Asn67, Val118 (mol A only), Phe120 (mol A only), and Asp121, while Coulombic interactions link the N^ζ^ atom of Lys7 (a component of the P_2_ subsite) and the polyphosphate chain, the closest approach being 3.6 Å to the β-phosphate in mol A.

**Table III:** Conformations of the Nucleotide Inhibitors

	5′-ATP		Ap_3_A		NADPH		NADP^+^	
	Mol A	Mol B	Mol A	Mol B	Mol A	Mol B	Mol A	Mol B
Torsion Angles (deg)^a^								
*N*-glycosidic bond								
O4′–C1′–N9–C4 (χ)	224	222	64	46	271	256	271	259
Conformational region	*anti*	*anti*	*syn*	*syn*	high-*anti*	high-*anti*	high-*anti*	high-*anti*
5′-substituents								
C5′–C4′–C3′–O3′ (δ)	130	106	110	106	146	136	146	137
O5′–C5′–C4′–C3′ (γ)	218	203	51	202	31	70	28	54
PA–O5′–C5′–C4′ (β)	90	102	191	125	143	197	153	168
O3A–PA–O5′–C5′ (α)	215	168	302	176	86^b^	53^b^	81^b^	73^b^
PB–O3A–PA–O5′ (ζ_p1_)	91	56	213	291	—	—	—	—
O3B–PB–O3A–PA (ζ_p2_)	224	299	309	238	—	—	—	—
PG–O3B–PB–O3A (ζ_p3_)	55	150	153	96	—	—	—	—
O3G–PG–O3B–PB (ζ_p4_)	293^b^	355^b^	196^b^	285^b^	—	—	—	—
2′-substituent								
P2′–O2′–C2′–C1′	—	—	—	—	130	140	131	139
P2′–O2′–C2′–C3′	—	—	—	—	244	254	246	256
Conformational Parameters								
Ribose pucker^c^								
*P* (deg)	140	352	106	19	177	147	182	149
Conformational region	C2′-*endo*	C2′-*exo*	O4′-*endo*	C3′-*endo*	C2′-*endo*	C2′-*endo*	C3′-*exo*	C2′-*endo*

a Torsion angle definitions follow IUPAC-IUB recommendations^42^ where possible and are illustrated in part in Figure 1.

b Because of rotational permutations, the positions of atom O3A in NADPH/NADP^+^ and atom O3G in 5′-ATP/Ap_3_A are ambiguous. However, they have been assigned as the most solvent-exposed oxygen and included for comparison.

c As described by Altona and Sandaralingam.^43^

**Table IV:** Potential Hydrogen Bonds Between RNase A and the Nucleotide Inhibitors

	Potential Hydrogen-Bonding Interactions (Protein Atom, Separation in Å)^a^							
	5′-ATP		Ap_3_A		NADPH		NADP^+^	
Inhibitor Atom	Mol A	Mol B	Mol A	Mol B	Mol A	Mol B	Mol A	Mol B
Adenine								
N1	N71 N^δ2^, 3.0	N71 N^δ2^, 3.0	–—	—	—	—	—	—
N6	N71 O^δ1^, 2.7	N71 O^δ1^, 2.8	N71 O^δ1^, 2.8	N71 O^δ1^, 3.2	(T70 O^γ1^, 2.9)^b^	—	(T70 O^γ1^, 3.0)^b^	—
N7	—	—	N71 N^δ2^, 3.1	N71 N^δ2^, 3.2	—	—	—	—
Ribose								
O2′	—	—	(T70 O^γ1^, 2.4)^b^	—	—	—	—	—
2′-phosphate								
OP1	—	—	—	—	H119 N^δ1^, 2.7	H119 N^δ1^, 2.7	H119 N^δ1^, 2.6	H119 N^δ1^, 2.7
OP2	—	—	—	—	H12 N^ɛ2^, 2.7	H12 N^ɛ2^, 2.7	H12 N^ɛ2^, 2.7	H12 N^ɛ2^, 2.7
	—	—	—	—	F120 N, 2.9	F120 N, 2.9	F120 N, 2.9	F120 N, 2.9
OP3	—	—	—	—	(Q11 N^ɛ2^, 3.4)^c^	Q11 N^ɛ2^, 3.3	(Q11 N^ɛ2^, 3.5)^c^	Q11 N^ɛ2^, 3.3
5′-α-phosphate								
O2A	—	—	H119 N^δ1^, 3.3	—	—	—	—	—
5′-β-phosphate								
O2B	Q11 O^ɛ1^, 3.1	—	H12 N^ɛ2^, 2.5	F120 N, 3.0	—	—	—	—
O3B	Q11 N^ɛ2^, 2.7	—	—	H12 N^ɛ2^, 3.2	—	—	—	—
5′-γ-phosphate								
O1G	H12 N^ɛ2^, 2.7	H12 N^ɛ2^, 2.7	K41 N^ζ^, 2.5	K41 N^ζ^, 2.6	—	—	—	—
	F120 N, 3.2	F120 N, 2.9						
O2G	H119 N^δ1^, 2.7	Q11 N^ɛ2^, 2.5	—	—	—	—	—	—
O3G	—	H119 N^δ1^, 2.7	—	—	—	—	—	—

a Potential hydrogen bonds are listed if identified with HBPLUS^44^ and corroborated by clear electron density for donor and acceptor.

b Crystal contact with symmetry-related mol B.

c Distance exceeds normal limits but is included for comparison.

5′-ATP is the first compound containing a γ-phosphate group for which the three-dimensional details of RNase A binding have been obtained. Previous studies on adenylic nucleotides that contain a 5′-pyrophosphate group (in various states of derivatization) are consistent in their description of a *syn* base conformation and in their placement of β-phosphate as the major P_1_ subsite ligand when bound to RNase A (Class II binding). The conformation observed here for RNase A-bound 5′-ATP represents a deviation from this pattern and offers an explanation for the stagnation in *K*_i_ encountered when moving from 5′-ADP to 5′-ATP. That is, although the Class II binding mode can support productive interactions of additional 5′-β-phosphate-linked substituents (e.g., in the binding of pdUppA-3′-p),^21^ with 5′-ATP these are not as favorable as those involving the γ-phosphate after adoption of the *anti* conformation observed here.

5′-ATP also inhibits EDN, albeit ∼25-fold less effectively (*K*_i_ = 21 μ*M*).^36^ The manner in which this compound binds to EDN (PDB entry 2C01)^24^ differs from that observed here with RNase A. A mixture of two conformations is present, neither of which is particularly well ordered. In one of these (conformation A), the adenine ring is bound as in RNase A, but the P_1_ subsite is occupied principally by the α-phosphate whereas the γ-phosphate is disordered. The catalytic centers of the two proteins are highly similar, and it seems that electronic and/or steric effects of a subtle nature are responsible for the differences in binding. Whatever the case, the superior inhibition of RNase A correlates with a better-defined and more extensive interaction between inhibitor and protein.

### Structure of the RNase A·Ap_3_A Complex

**FIGURE 4:**
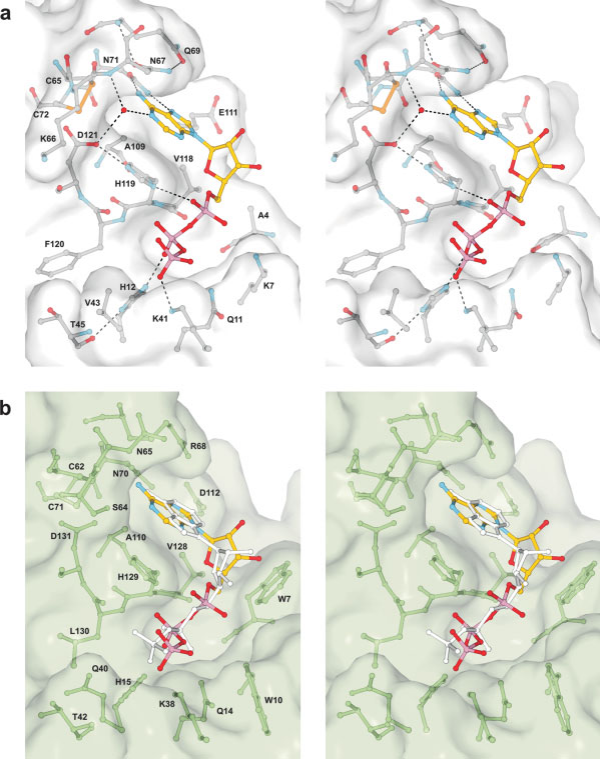
RNase A·Ap_3_A complex (mol A). (a) Enzyme and inhibitor in the same representation and orientation as in Figure 3. The inhibitor is disordered beyond the 5′-γ-phosphate. (b) Comparison with the EDN·Ap_3_A complex (PDB entry 2C02).^24^ The two complexes were superposed on the basis of the C^α^ positions of key nucleotide-binding residues (from RNase A, mol A: Q11, H12, K41, T45, H119, and F120; from EDN: Q14, H15, K38, T42, H129, and L130). Shown in stereo in the same orientation as in panel a are ball-and-stick representations of RNase A-bound Ap_3_A (colored as in panel a), EDN-bound Ap_3_A (white), and neighboring EDN residues (green), along with a surface representation of EDN. Main chain N, C, and O atoms of residues W10, Q14, H15, K38, Q40, and D112 are omitted for clarity. The EDN-bound inhibitor is disordered beyond the 5′-β-phosphate.

Extension of 5′-ATP by attachment of a 5′-linked adenosine, thereby generating Ap_3_A (Figure 1), causes a 34-fold decrease in affinity (*K*_i_ = 29 μ*M*).^36^ To find an explanation, we determined the structure of the RNase A·Ap_3_A complex. The electron density map indicates the position of a portion of the nucleotide corresponding to a 5′-ATP moiety (Figure 2b), and the remainder of the inhibitor is presumed to be in no fixed conformation. The visible portion has the adenine/His119 rotamer A stacking arrangement and *syn* base conformation of the Class II binding mode associated with 5′-pyrophosphate-containing adenine nucleotides (Figure 4a; Table III). There are modest differences in the binding of the inhibitor to the two protein chains in the asymmetric unit. These appear to derive from a hydrogen bond in mol A between O2′ of the ribose and O^γ1^ of a symmetry-related ThrB70 residue. This has negligible impact on the adenine position but alters the conformation of the ribose and, to a lesser degree, the polyphosphate chain (Table III). Two hydrogen bonds between the adenine and the side chain of Asn71 are maintained, as is one between the β-phosphate and His12, and one between the γ-phosphate and Lys41 (Table IV). The N^ζ^ atom of Lys7 is ∼ 4 Å away from the α-phosphate, close enough for significant Coulombic interactions. Differences between the two instances of the inhibitor include a hydrogen bond between the α-phosphate and His119 in mol A only and one between the β-phosphate and Phe120 in mol B only.

Although they comprise the same set of atoms, RNase A-bound 5′-ATP and the ordered portion of likewise-bound Ap_3_A exhibit conformations that are clearly very different from one another. In the 5′-ATP complex, the compact conformation of the polyphosphate chain and the tendency of the γ-phosphate toward burial among the P_1_ subsite residues appears to leave little scope for the addition of a second 5′-linked adenosine (see Figure 3). A switch to the Class II binding mode, however, provides an exit route for the 5′-extension, as is observed for other inhibitors such as pdUppA-3′-p.^21^ The loss of 1–2 hydrogen bonds that accompanies this change most likely contributes to the decrease in affinity that is observed when moving from 5′-ATP to Ap_3_A.

Ap_3_A inhibits EDN with a similar potency (*K*_i_ = 70 μ*M*).^36^ When bound to EDN (PDB entry 2C02),^24^ the conformation of the second adenosine moiety is likewise disordered, but a *syn* base conformation is not observed. The reason for this is the need to avoid an overlap between the O3′ edge of the ribose and the side chain of Trp7 (a residue whose counterpart in RNase A is Ala4) (Figure 4b). The α- and β-phosphates appear to hydrogen-bond with EDN's P_1_ subsite but it was not possible to arrive at a definitive arrangement due to disorder in the nucleotide.^24^ It seems likely that hydrogen-bonding is less extensive than in the RNase A complex and that this is compensated for by the adoption of a more favorable base conformation to give a similar binding affinity.

### Structures of the RNase A·NADPH and RNase A·NADP^+^ Complexes

The compound ppA-2′-p is among the tightest-binding RNase A inhibitors known (*K*_i_ = 0.52 μ*M*).^18^ NADPH and NADP^+^ are naturally-occurring substances that contain a ppA-2′-p moiety yet are considerably less effective inhibitors with *K*_i_ values of 12 and 63 μ*M*, respectively.^36^ Seeking an explanation, we determined the crystal structures of NADPH and NADP^+^ in complex with RNase A. These reveal no major differences between the binding of the two inhibitors and therefore they will be treated together.

**FIGURE 5:**
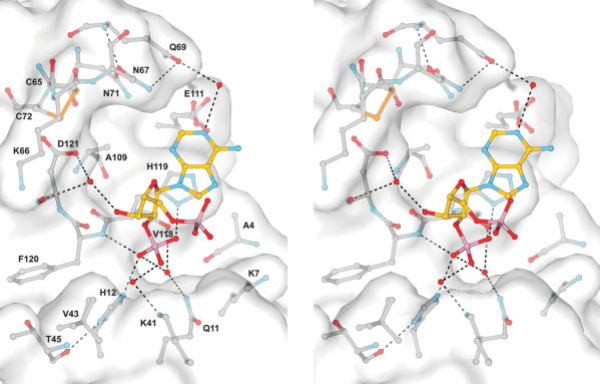
RNase A·NADPH complex (mol A). Shown are enzyme and inhibitor in the same representation and orientation as in Figure 3. The side chains of V43 and E111 are drawn in dual conformation (as fitted to the electron density) whereas the side chain of V118 is omitted for clarity. The inhibitor is disordered beyond the 5′-α-phosphate.

The electron density map permits modelling of a pA-2′-p moiety; no density is observed for the 5′-β-phosphate, nicotinamide or the intervening sugar and they are presumed to be in no fixed conformation (Figures 2c and 2d). The adenine ring stacks against His119 (rotamer B) and the 2′-phosphate occupies the P_1_ subsite (Figure 5). This conforms to Class III binding and matches the mode reported for pA-2′-p bound to crystalline RNase A (mol A) and EDN or to Ang in solution.^22^,^23^,^45^ Again, there are small conformational differences between the binding of the inhibitor to each of the two protein chains in the asymmetric unit. These appear to stem from a hydrogen bond in mol A between adenine atom N6 and atom O^γ1^ of a symmetry-related ThrB70 residue (the same residue that affects Ap_3_A binding). This brings about a 3.4-Å shift in the position of atom N6 and compensatory adjustments to the remainder of the inhibitor (Table III). However, it has minimal effect on the 2′-phosphate group, which is particularly well ordered in the electron density map, suggesting that it is the main focal point for binding. The 2′-phosphate hydrogen-bonds with His12, His119, Phe120, and Gln11 (mol B only) (Figure 5, Table IV). There are also water-mediated interactions between the inhibitor and Gln11, Lys41, Gln69, Asp121, Val118 (mol A only), and Asn71 (mol B only). The N^ζ^ atom of Lys7 is roughly equidistant from the 2′- and 5′-α-phosphate groups; at 5.8–6.6 Å away, it is somewhat more remote than in the other complexes described in this report. The 5′-α-phosphate makes no hydrogen-bonding interactions with the protein and is oriented toward the solvent, indicating that the nicotinamide nucleotide portion is in a disordered, solvent-exposed state.

Given the similarity between the interactions of RNase A (mol A)-bound pA-2′-p and those of the visible portions of NADPH/NADP^+^, one might expect a correspondence in *K*_i_ values. The literature contains no inhibition constant for the RNase A·pA-2′-p interaction, but structural and kinetic data on EDN,^23^ ECP,^25^ and Ang^45^,^46^ indicate that it will be in the region of that measured for pA-3′-p (*K*_i_ = 4.7 μ*M*),^18^ which is indeed similar to that reported for NADPH. The present data do not provide a structural basis for the 5-fold decrease in affinity that is measured when moving from NADPH to NADP^+^. It seems most likely that this is related to electrostatic repulsion of the cationic pyridinium ring of NADP^+^ by the surplus of positive charge in the active site of the enzyme, as suggested previously.^36^

Although both NADPH and NADP^+^ contain ppA-2′-p as a structural element, the adenylic portions of these dinucleotides do not adopt the *syn* base (Class II) conformation observed when ppA-2′-p binds to RNase A in isolation.^20^ It is possible that the position and orientation observed for the 5′-β-phosphate group of enzyme-bound ppA-2′-p leave no room for accommodation of an adjacent ribosyl group. This differs from the corresponding portion of enzyme-bound ppA-3′-p^20^ which has been shown to offer access to additional 5′-linked substituents, e.g., those in pdUppA-3′-p.^21^

### Structure of the RNase A·PP_i_ Complex

**FIGURE 6:**
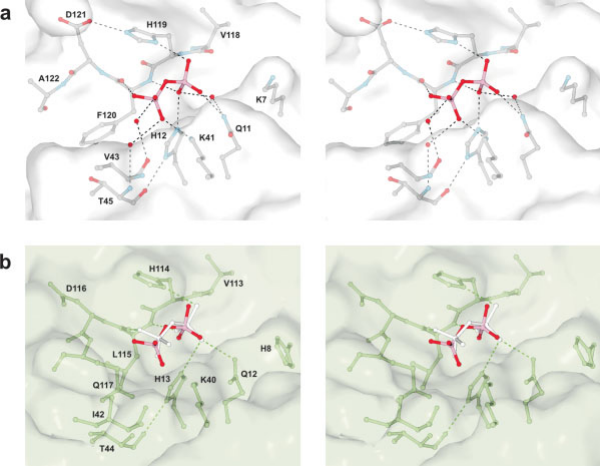
RNase A·PP_i_ complex (mol B). (a) Enzyme and inhibitor in the same representation and approximately the same orientation as in Figure 3. (b) Comparison with the Ang·PP_i_ complex (PDB entry 1H52).^49^ The two complexes were superposed on the basis of the C^α^ positions of key nucleotide-binding residues (from RNase A, mol B: Q11, H12, K41, T45, H119, and F120; from Ang: Q12, H13, K40, T44, H114, and L115). Shown in stereo from the same viewpoint as for panel a are ball-and-stick representations of RNase A-bound PP_i_ (colored as in panel a), Ang-bound PP_i_ (white), and neighboring Ang residues (green), along with a surface representation of Ang. Main chain N, C, and O atoms of residues H8, Q12, H13, and K40 are omitted for clarity. Dashed lines denote hydrogen bonds.

A pyrophosphate moiety is an invariant substituent of the most potent nucleotide-based inhibitors identified to date. Even as a free ion, it inhibits RNase A appreciably (*K*_i_ ≈ 170 μ*M*),^47^ highlighting its contribution to the affinity of these inhibitors. In the crystal structure of the RNase A·PP_i_ complex, electron density for the ion is clear, particularly so in mol B (Figure 2e). Both halves of the ion interact with the protein via multiple direct and water-mediated hydrogen bonds (Figure 6a). There is very little difference between the two instances of the ion in the asymmetric unit. One half of the ion (which we designate the α-phosphate) hydrogen-bonds with Gln11 N^ɛ2^, His12 N^ɛ2^, His119 N^δ1^, and Phe120 N and experiences Coulombic attraction to Lys 7 N^ζ^, which is at a distance of 5.2 Å. The other half (the β-phosphate) hydrogen-bonds with Lys41 N^ζ^ and Phe120 O (provided that the relevant PP_i_ oxygen is protonated). Water-mediated hydrogen bonds are also made with Gln11 N^ɛ2^ (α-phosphate), Val43 O (β-phosphate, mol B only), Thr45 N (β-phosphate), and Val118 O (α-phosphate, mol A only).

The position of the α-phosphate is a good match to that of a conventionally-bound 5′-α-phosphate such as that present in pA-3′-p or d(CpA)^22^,^34^ and is an approximate match to that of the 5′-β-phosphate present in ppA-3′-p, ppA-2′-p or 3′,5′-pyrophosphate-linked inhibitors such as pdUppA-3′-p.^20^,^21^ The position of the β-phosphate does not correspond well with that of any phosphate group among known inhibitors. It is closest to that of the 5′-γ-phosphate in the Ap_3_A complex (Figure 4a) but is further toward the B_1_ subsite.

The details of the RNase A·PP_i_ structure aid in the interpretation of the nucleotide-binding properties of the homologous ribonuclease, Ang. Inhibitors of Ang have potential therapeutic use as anti-cancer drugs (reference^8^ and others therein) but their binding has proved to be far less amenable to crystallographic study owing to the obstructive position of symmetry-related molecules in all crystal forms identified to date and, in some cases, to the cryptic nature of Ang's B_1_ subsite.^48^ The largest entity that has been observed bound to the active site of Ang is a PP_i_ ion.^49^ This binds in a position that is very similar to that observed in the RNase A·PP_i_ complex, with Ang residues Gln12, His13, His114, and Leu115 substituting for RNase A residues Gln11, His12, His119, and Phe120, respectively (Figure 6b). The main difference between the two complexes is the rotameric state of the β-phosphate, which permits a hydrogen bond with Lys41 in RNase A but not with its counterpart (Lys40) in Ang. The most likely cause is the need to avert a clash with the C^γ^ atom of Gln117 (a component of the novel C-terminal segment of Ang) which, when superposed, is located ∼ 2.5 Å from the position occupied by one of the β-phosphate oxygens in the RNase A complex. The loss of this hydrogen bond may contribute to the lower affinity of PP_i_ for Ang (*K*_i_ = 1.7 m*M*).^49^

## DISCUSSION

### Use of Monoclinic RNase A Crystals in the Study of Inhibitor Binding

Since their discovery,^20^ monoclinic RNase A crystals have proven to be well suited to the study and development of ribonuclease inhibitors. In their favor, they grow at low ionic strength and moderate acidity, both of which are conducive to nucleotide binding.^16^ They also offer good access to the whole B_1_-P_1_-B_2_ region of each of the protein molecules in the asymmetric unit, as indicated by the excellent electron density observed for enzyme-bound pdUppA-3′-p.^21^ The quality of the present structures supports the utility of these crystals but reaffirms one drawback, namely the potential impact of symmetry-related protein chains on the binding of inhibitors to the B_2_ subsite. Mol A is the most affected owing to potential direct hydrogen-bonding interactions between the inhibitor and a symmetry-related ThrB70 residue and, to a lesser degree, to constrainment of GluA111 by its packing against a symmetry-related TyrB115 residue. In addition, GlnA69 and GlnB69 pack against one another, which could conceivably constrain either residue. These features do not affect the overall binding mode or conformational order of the present inhibitors but they have been seen to do so, for example in the binding of pA-2′-p and dUppA.^22^,^27^ In those cases where there is a degree of conformational disorder and an ensemble of inhibitor conformations exists (e.g., the binding of Ap_3_A, NADPH or NADP^+^), the symmetry-related contacts of mol A might be viewed as a helpful means of selecting a representative conformation, but it may be that mol B is a more reliable indicator of solution behavior.

### Prospects for Improved Adenylic Inhibitors of RNase A

The conformational diversity of RNase A-bound adenylic inhibitors continues to expand. With our report of the RNase A·5′-ATP structure, there are now accounts of the binding of 5′-α-, 5′-β-, 5′-γ-, and 2′-phosphates to the P_1_ subsite. The torsional flexibility of the nucleotides and the adaptability of the B_2_ subsite are key factors. Despite the array of possible conformations, the larger inhibitors investigated here (Ap_3_A, NADPH, and NADP^+^) are only partially visible in their respective electron density maps. The possibility of nonenzymatic cleavage of these inhibitors must be considered. Ap_3_A is known to be susceptible to hydrolytic cleavage within the polyphosphate chain but the reaction yields products (5′-ADP and 5′-AMP) that are smaller than the visible moiety (5′-ATP) and occurs at an insignificant rate (*t*_½_ = 154 d at 60°C, pH 6.0, and *I* = 0.1).^50^ For pyridine nucleotides under mildly acidic conditions, polyphosphate cleavage is not a significant form of degradation. It does occur with oxidized forms such as NADP^+^ but elevated temperatures and alkaline conditions are required for the detection of products.^51^,^52^ In view of this, the partial electron density observed in the present complexes most likely reflects a lack of interaction between one half of the dinucleotide and the protein. It appears that, as the complexity of the 5′-linked extension increases, the need to avoid unfavorable protein·inhibitor contacts becomes an overiding influence on the binding mode and a reduced number of modes can support the binding of the compound.

The incomplete interaction of Ap_3_A, NADPH, and NADP^+^ with RNase A is reminiscent of the binding of Ap_3_A and Ap_4_A to EDN.^24^ With the latter enzyme, further extension of the polyphosphate linker to form Ap_5_A brought about a far more extensive interaction, indicating that inclusion of an appropriate linker can have a dramatic effect on binding. Hence, it remains possible that the decreases in the *K*_i_ for RNase A inhibition encountered when moving from Ap_3_A (*K*_i_ = 29 μ*M*) through Ap_4_A (*K*_i_ = 2.6 μ*M*) to Ap_5_A (*K*_i_ = 0.23 μ*M*) reflect the formation of new interactions permitted by linker extension, as previously suggested.^36^

The details of the NADPH and NADP^+^ complexes give insight into the suitability of pA-2′-p or ppA-2′-p as a core for the development of larger inhibitors of RNase A. Although three different binding modes have been observed for 2′-phosphoadenosine-based nucleotides (Table I), none of these permits binding of the nicotinamide portion of the present nucleotides. As with the diadenosine polyphosphates, the inclusion of extended, unbranched linkers may be necessary to facilitate additional interactions beyond the 5′-β-phosphate.

### Inhibitors of Specific Ribonuclease Subclasses

The development of inhibitors that are specific for individual ribonuclease subclasses would be of great value to the study of these enzymes in vivo and for the development of potential ribonuclease-directed therapeutics. High-throughput screening has identified several non-nucleotide inhibitors that bind slightly more tightly to Ang than to RNase A.^53^ Existing adenylic inhibitors, on the other hand, tend to select against Ang, for which they exhibit *K*_i_ values that are 2–3 orders of magnitude greater than those obtained for RNase A.^16^ With the nucleotides, the design process has been hampered by an inability to obtain Ang·inhibitor complexes in crystalline form and it has been necessary to extrapolate from data on RNase A and other enzymes. To help test the validity of these measures, a direct comparison can now be made between the RNase A·PP_i_ and Ang·PP_i_ structures.^49^ It is clear that the manner in which PP_i_ binds to RNase A is not an accurate indicator of what occurs when the same group is O5′-linked to an adenylic nucleotide. The same is true for Ang, as can be deduced from heteronuclear NMR data on its interaction with ppA-2′-p and dUppA-2′-p.^45^ Although there is a close correspondence in the ligand position in the RNase A·PP_i_ and Ang·PP_i_ structures, there is a difference in the manner in which the two enzymes bind ppA-2′-p (Class II and Class III modes, respectively). Nevertheless, with the larger derivatives that have been examined (NADPH/NADP^+^ with RNase A and dUppA-2′-p with Ang), the Class III mode prevails (as does the nonengagement of the second nucleotide), suggesting that similar strategies may be necessary to improve the affinity of 2′-phosphoadenosine-based nucleotides for these enzymes.

It has been suggested that side chain differences located toward the N-terminus of the protein could contribute to differences in the affinity of adenylic inhibitors for various ribonucleases.^17^ The contrast between the RNase A·Ap_3_A and EDN·Ap_3_A structures supports this view (Figure 4b). The complete conformational switch that is apparently triggered by the Trp7 side chain of EDN highlights that such steric effects can be strong and could be exploited in the inhibitor design process. In particular, the close proximity of Trp7 to the ribosyl O3′ atom of the inhibitor suggests that O3′-linked substituents above a certain size may not be tolerated well by this enzyme and may disfavor binding.

## MATERIALS AND METHODS

### Crystallization and Data Collection

RNase A and the sodium salts of 5′-ATP, Ap_3_A, NADPH, NADP^+^, and PP_i_ were obtained from Sigma and dissolved in water. RNase A crystals were grown at 16°C by the hanging-drop/vapor-diffusion method as described previously.^20^ These were transferred to a stabilizing solution (25% PEG 4000, 0.02*M* sodium citrate buffer, pH 5.5) supplemented with either 100 m*M* 5′-ATP, 100 m*M* Ap_3_A, 20 m*M* NADPH, 20 m*M* NADP^+^, or 100 m*M* PP_i_ at 16°C for 24 h. Diffraction data were collected at 100 K; for crystals soaked in 5′-ATP, NADPH, NADP^+^, and PP_i_, this was carried out on stations 14.1, 9.6, 9.6, and 14.2 of the SRS, Daresbury, UK, respectively, whereas for crystals soaked in Ap_3_A, this was conducted in-house using a MAR Research 30-cm image plate mounted on a Rigaku RU-H3R Cu *K*α radiation source. All data were processed using the HKL suite^54^ and TRUNCATE.^55^,^56^ Detailed data processing statistics are given in Table II.

### Phase Determination and Refinement

Initial phases were determined using the molecular replacement routines of AMoRe^57^ with reference to coordinates of unliganded RNase A obtained previously under the same crystal growth conditions (PDB entry 1AFU).^20^ Refinement was conducted initially using the rigid-body, simulated annealing, coordinate minimization, and *B*-factor refinement elements of CNS^58^ and latterly using the maximum-likelihood routines of REFMAC5.^39^ Throughout, 5–10% of reflections (depending on resolution) were set aside for cross-validation.^37^ After every refinement cycle, electron density maps were calculated and used to guide manual adjustments made with either O or COOT.^59^,^60^ Ligands or fragments thereof were introduced on the basis of m*F*_o_-D*F*_c_ maps and refined using geometrical restraints provided by the CCP4 monomer library.^61^ Water molecules were added at positions where m*F*_o_-D*F*_c_ electron density peak heights exceeded 3σ and where potential hydrogen bonds could be made. Model validation was conducted with MolProbity.^40^ Detailed statistics for each model are given in Table II.

Data Deposition: The coordinates and structure factors of RNase A in complex with 5′-ATP, Ap_3_A, NADPH, NADP^+^, and PP_i_ have been deposited in the Protein Data Bank under accession numbers 2W5G, 2W5I, 2W5K, 2W5L, and 2W5M, respectively.
